# Evaluating the Effect of Window-to-Wall Ratios on Cooling-Energy Demand on a Typical Summer Day

**DOI:** 10.3390/ijerph18168411

**Published:** 2021-08-09

**Authors:** Jiayu Li, Bohong Zheng, Komi Bernard Bedra, Zhe Li, Xiao Chen

**Affiliations:** 1 School of Architecture and Art, Central South University, Changsha 410083, China; J.Y.Li@csu.edu.cn (J.L.); zhengbohong@csu.edu.cn (B.Z.); komibedra@csu.edu.cn (K.B.B.); 2 College of Landscape Architecture and Art Design, Hunan Agricultural University, Changsha 410128, China

**Keywords:** Envi-met, TRNSYS, window-to-wall ratio, energy demand

## Abstract

The window-to-wall ratio (WWR) significantly affects the indoor thermal environment, causing changes in buildings’ energy demands. This research couples the “Envi-met” model and the “TRNSYS” model to predict the impact of the window-to-wall ratio on indoor cooling energy demands in south Hunan. With the coupled model, “Envi-met + TRNSYS”, fixed meteorological parameters around the exterior walls are replaced by varied data provided by Envi-met. This makes TRNSYS predictions more accurate. Six window-to-wall ratios are considered in this research, and in each scenario, the electricity demand for cooling is predicted using “Envi-met + TRNSYS”. Based on the classification of thermal perception in south Hunan, the TRNSYS predictions of the electricity demand start with 30 °C as the threshold of refrigeration. The analytical results reveal that in a 6-storey residential building with 24 households, in order to maintain the air temperature below 30 °C, the electricity required for cooling buildings with 0% WWR, 20% WWR, 40% WWR, 60% WWR, 80% WWR, and 100% WWR are respectively 0 KW·h, 19.6 KW·h, 133.7 KW·h, 273.1 KW·h, 374.5 KW·h, and 461.9 KW·h. This method considers the influence of microclimate on the exterior wall and improves the accuracy of TRNSYS in predicting the energy demand for indoor cooling.

## 1. Introduction

The residential energy consumption in China accounts for about 21% of China’s total energy consumption [1,2], and 70% of that energy is consumed for the purpose of modifying the indoor thermal environment [3]. In China, the peak energy consumption in residential buildings generally occurs during summers [4,5], and this trend has been intensified along with global warming [6,7]. In urban planning and building science, the window-to-wall ratio (WWR) is a spatial parameter usually determined by aesthetic, energy-saving, and daylight considerations [8,9]. In this study, we mainly discuss the influence of WWR on energy savings. WWR significantly affects the indoor thermal environment [10,11,12], particularly the indoor energy demand during summer [13,14]. In urban planning, most WWR-related research focuses on its impact on the thermal environment at the urban scale [15,16], without considering its impact on building energy savings. In urban planning, the WWR is investigated in the urban environment rather than in isolated buildings. In building science, although many studies have researched how WWR influences the building energy demand [17,18,19], most of those have been conducted on single buildings, without considering microclimate factors, which greatly affect the indoor air temperature [20,21].

There are also some differences in the research technologies associated with WWR in urban planning and building science. In urban planning, WWR studies are mostly conducted using CFD software (Computational Fluid Dynamics) [22,23,24], which is capable of simulating the thermal behavior of various materials under various morphological conditions, calculating their influences on local wind speed, air temperature, and relative humidity [25,26,27]. Nevertheless, the CFD-based software packages are not advanced in their ability to calculate energy consumption. The Transient Systems Simulation program (TRNSYS) is widely used by architects to predict building energy demands [28,29,30], but these TRNSYS programs are not CFD-based programs, leading to the following deficiencies. First, the TRNSYS program does not consider wind speed changes around the exterior walls, but instead only considers a fixed value, which causes inaccuracies in the energy demand predictions because of the strong effect of wind speed on the convective heat transfer from building surfaces [31,32,33]. Furthermore, the TRNSYS program cannot calculate the air temperature around the exterior walls for which it also considers a fixed air temperature value [31,34]. Overall, TRNSYS calculates the indoor cooling demand without considering the changes in wind speed and air temperature caused by the urban microclimate.

Unlike other CFD-based software, Envi-met incorporates thermodynamics principles, allowing the model to also analyze short- and long-wave radiations and plant transpirations. “Envi-met” has thus been selected to simulate the microclimate around the exterior walls first, then its output is used to improve the accuracy of the energy demand prediction by “TRNSYS”.

In fact, the effect of WWR on building cooling energy demand is also influenced by the window-to-floor ratios. Because the residential buildings in China are commercial houses with little changes in floor area, only WWR is discussed in this study. The building models are built according to the size of actual residential buildings in southern Hunan. Six WWR scenarios are constructed to investigate their varied effects on the indoor energy demand. There is no standard threshold for cooling-energy initiation, which is affected by the body’s tolerance to temperature, and Hunan people sense 30 °C as the boundary temperature between slightly warm and warm, therefore, 30 °C has been adopted as the threshold of refrigeration start-up in this study.

## 2. Methods and Parameters

### 2.1. Research Model

**Figure 1:**
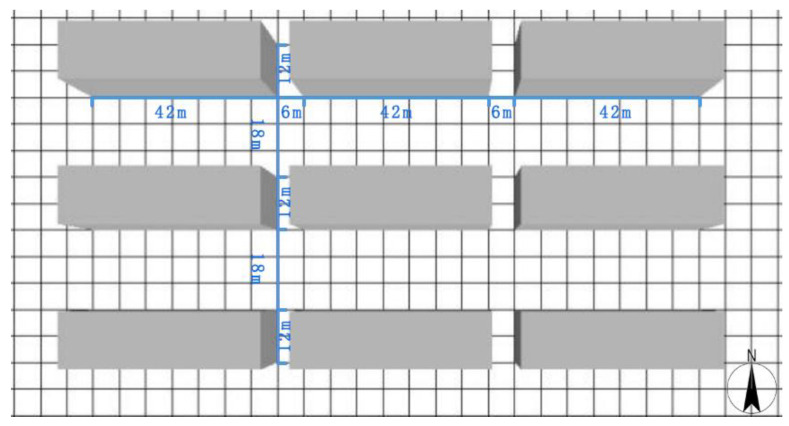
The typical residential unit in southern Hunan.

Among all the building types, residential buildings consume the largest proportion of cooling and heating energy [35]. Hence, residential buildings have been selected as the building type in this study. Furthermore, southern Hunan is a typical region in China with hot summers and cold winters. This entails a high energy demand for cooling during summer. In this study, a typical residential block in southern Hunan was chosen as the research target. The advocated area for commercial housing in China is 90–144 m^2^/household, and 126 m^2^ (10.5 m × 12 m) has been adopted in this research. Additionally, the layout of buildings in the residential block respects China’s national “standard for planning and designing urban residential areas” (GB 50180-2018) [36], where the distance between buildings in the east–west direction should not be less than 6 m as a fire safety measure. The distance between the buildings in the south–north direction shall comply with the requirement of a sunshine coefficient (1:1). Generally, for the optimum exploitation of urban land, the actual construction is done according to the fire safety and building sunshine required boundaries. The typical layout of a residential block in southern Hunan is shown in Figure 1.

**Figure 2:**
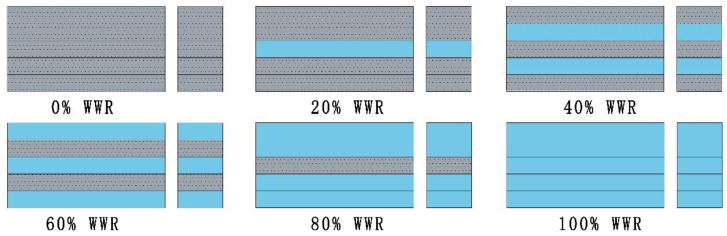
The six window-to-wall ratio (WWR) scenarios in this study.

Six scenarios of WWR are constructed in Figure 1. The six scenarios of WWR are 0%, 20%, 40%, 60%, 80%, and 100%, as shown in Figure 2.

In the study, clear float glass, a commonly used glass in southern Hunan, has been adopted. Additionally, the walls are constructed with concrete. The properties of the glass and concrete used in this research are presented in Table 1 [37,38].

### 2.2. The Setting of Meteorological Data

**Figure 3:**
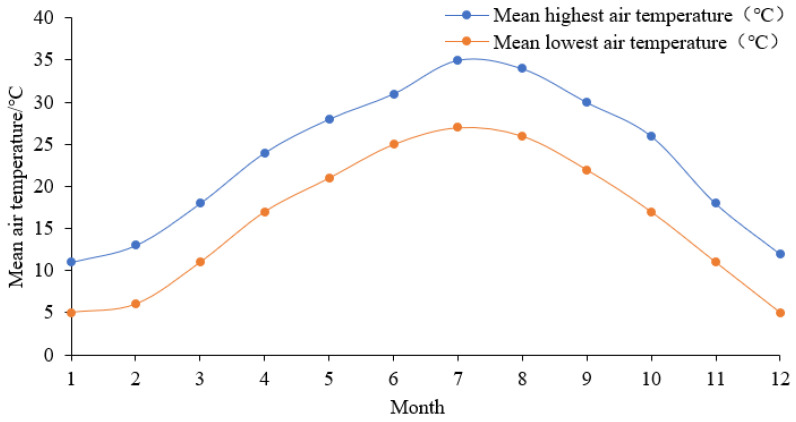
The annual variations of the air temperature in southern Hunan.

The meteorological data of southern Hunan were collected from the Chenzhou Meteorological Bureau. This study analyzes the meteorological data of the past ten years, including the averages of the maximum and minimum air temperatures of each month. The annual variations of the maximum and minimum air temperatures are presented in Figure 3. Figure 3 indicates that the hottest month in southern Hunan appears in July, with the average maximum and minimum air temperatures being 35 °C and 27 °C, respectively.

**Figure 4:**
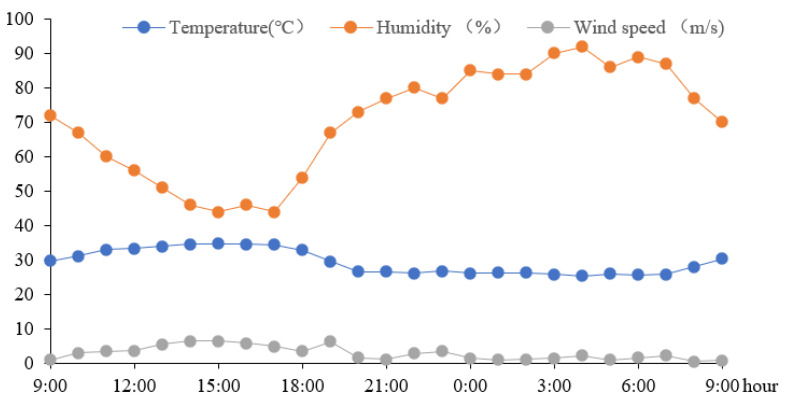
The daily variations of the air temperature, humidity, and wind speed.

Reviewing the hourly meteorological data over the past ten years, the meteorological characteristics of 25 July 2017 appear to be representative of the July conditions over recent years. Furthermore, from 9:00 on 25 July 2017 to 9:00 on 26 July 2017, southern Hunan was sunny. Therefore, the period from 9:00 on 25 July 2017 to 9:00 on 26 July 2017 was determined to be a typical summer day for this research. The daily variations of air temperature, humidity, and wind speed are presented in Figure 4, which are used as boundary conditions for the following simulations.

### 2.3. The Calculation of the Cooling-Energy Demand Caused by WWRs

**Figure 5:**
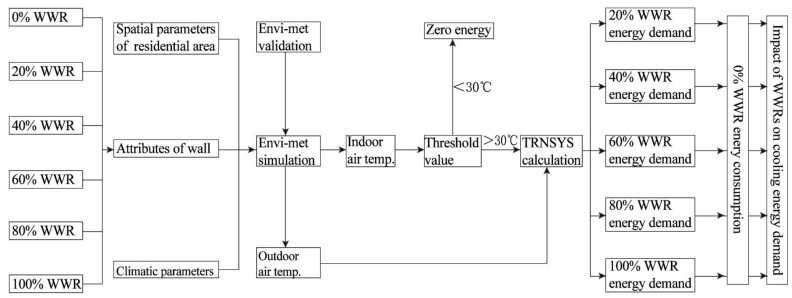
The technical framework of the study.

From a methodological perspective, coupling the CFD-based software Envi-met with the Transient Systems Simulation program TRNSYS fills in the gap between urban planning and building science [39]. The implication of WWR on the energy demand mainly originates from the fact that WWR modifies the indoor and outdoor thermal environment, changing the cooling load necessary for maintaining indoor thermal comfort [40]. By replacing the fixed outdoor boundary condition in TRNSYS with accurate microclimate data provided by Envi-met, the coupled “Envi-met + TRNSYS” improves the accuracy of energy demand estimations. This method also makes up for the defect caused by TRNSYS calculating the indoor air temperature without considering the convective heat transfer affected by wind speed on the exterior walls. The technical framework of this study is shown in Figure 5.

The modeled residential block is located at 25°74′ N, 112°96′ E. The meteorological data in Figure 4 are used as boundary conditions for the Envi-met simulation. The physical parameters of the wall and glass used in the model are shown in Table 1. The indoor and outdoor temperatures are simulated under the six WWR scenarios for 24 h, from 9:00 on 25 July 2017 to 9:00 on 26 July 2017. The Envi-met output data are used as TRNSYS input for cooling load prediction.

Three core computational models are involved in this methodology: the Envi-met model for the simulation of the indoor and outdoor thermal environments, the threshold criteria for cooling-energy initiation, and the TRNSYS model for cooling energy demand prediction.

#### 2.3.1. The Simulation of the Indoor and Outdoor Thermal Environment

Envi-met is designed to simulate the interactions between surfaces, plants, and the air in a city [41,42]. Urban planners are interested in the impact of urban parameters on the overall indoor thermal environment [43]. Among all the simulation models, CFD-based models are preferred by urban planners for microclimate consideration, and Envi-met model is ideal for studying indoor thermal environments. Envi-met estimates the indoor air temperature from the heat convection on the interior surface of the walls and roofs, and also from the energy transmitted through transparent glass [44]. The calculation of indoor air temperature ($T_{i}^{*}$) by Envi-met is done according to the following formula [45]:

(1) $$T_{i}^{*}=T_{i}+\frac{1}{C_{p}V}\int_{e=1}^{E}A\left(e\right)\left(Q_{sw}^{tr}\left(e\right)+h_{c,i}\left(T_{3}^{*}\left(e\right)-T_{i}\right)\right)dt$$

In this equation, $T_{i}$ is the original air temperature in zone *i*, $C_{p}$ indicates the specific heat capacity of air, which is 1.003 J(kg·K)^−1^, *V* represents the volume of the building zone *i*, and $T_{i}^{*}$ indicates the new air temperature after a time dt. *E* is the number of façades constituting the zone of *i*, and *A*(*e*) is employed to indicate the surface area of zone *i*. $Q_{sw}^{tr}$ indicates the short-wave radiation transmitted into zone *i* though the transparent façade *e*, and $h_{c,i}$ is the heat convection coefficient that calculates the sensible heat transfer between the inner walls and ambient air. 

**Figure 6:**
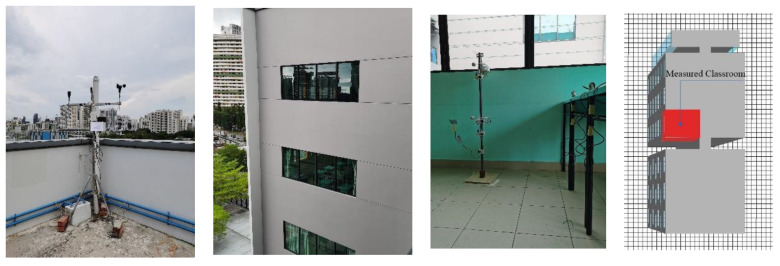
The settings of the field measurement and Envi-met model.

Many researchers have confirmed the accuracy of the Envi-met model in simulating indoor and outdoor thermal environments [46,47,48]. In this study, the accuracy of the Envi-met model has also been validated by a field measurement. The measurement was carried out at Alexandra Primary School (103°49′25.78″ E, 1°17′29.15″ N) from 18 March to 19 March 2021. The settings of the field measurement and Envi-met model are shown in Figure 6.

**Figure 7:**
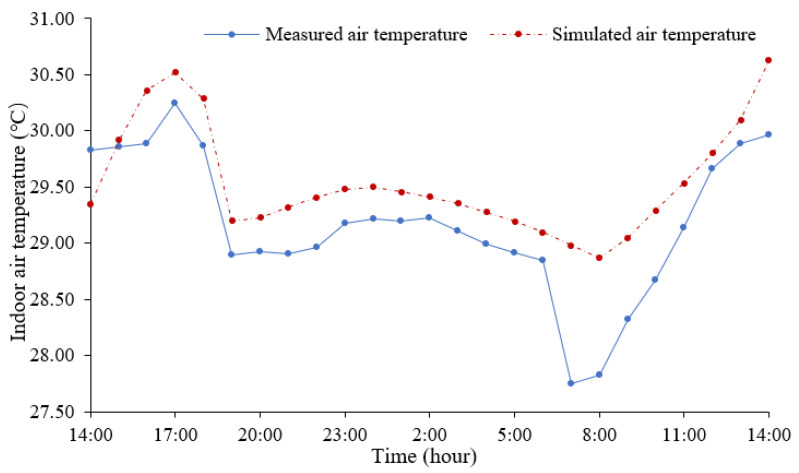
The measured and simulated indoor air temperatures.

The measured and simulated indoor air temperatures are shown in Figure 7, where the solid line represents the measured indoor air temperatures, and the dotted line indicates the simulated indoor air temperatures. The *R^2^* of the measured and simulated indoor air temperatures is 0.902. This field measurement confirms that the Envi-met model is reliable for the prediction of the indoor and outdoor thermal environments in the context of this study.

#### 2.3.2. Threshold Standard for Cooling-Energy Initiation

There is no unified temperature for cooling-energy initiation, which is determined by the body’s tolerance to temperature, and the temperature tolerance of the human body is affected by each individual’s thermal sensation, thermal preference, demographic background, clothing, and activity level [49]. Therefore, citizens of different regions have different thermal acceptability levels. Many cities such as Singapore and Hong Kong have investigated the thermal acceptability of their respective citizens [50,51]. Liu Weiwei et al. investigated the thermal perceptions of residents of Hunan [52]. According to their investigation, Hunan people sense 30 °C as being the boundary temperature between slightly warm and warm. This study, therefore, considers 30 °C as the threshold value to assess the indoor cooling load and energy demand.

#### 2.3.3. Calculation of Energy Demand by TRNSYS

**Figure 8:**
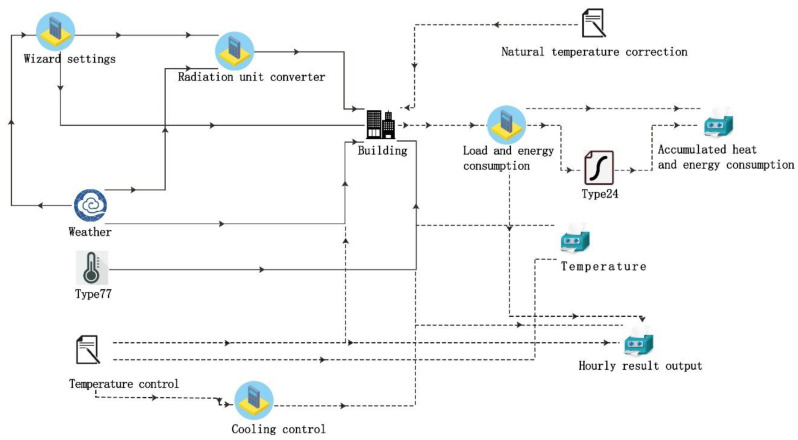
The schematic diagram of the TRNBuild module.

TRNSYS is a simulation tool used to simulate the energy load in a climatic environment. Its model is configured based on the building code certified by the United States Department of Energy [53]. Particularly, TRNSYS can simulate the transient effect of thermal mass, the heat transfer, etc. In this study, The TRNBuild of TRNSYS version 18 has been applied to simulate the cooling load and the electricity demand using indoor and outdoor air temperatures provided by Envi-met. The schematic diagram of the TRNBuild module is shown in Figure 8.

## 3. Results and Discussion

### 3.1. Indoor Air Temperatures Regulated by WWRs

**Figure 9:**
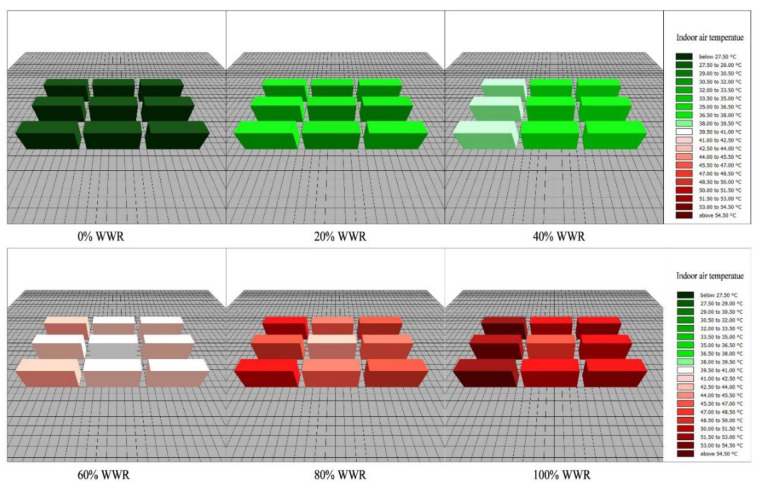
A sample of simulated indoor air temperatures.

The Envi-met model simulates the indoor air temperatures throughout the day, and 24-h indoor air temperatures are extracted. A group of simulated indoor air temperatures is shown in Figure 9.

**Figure 10:**
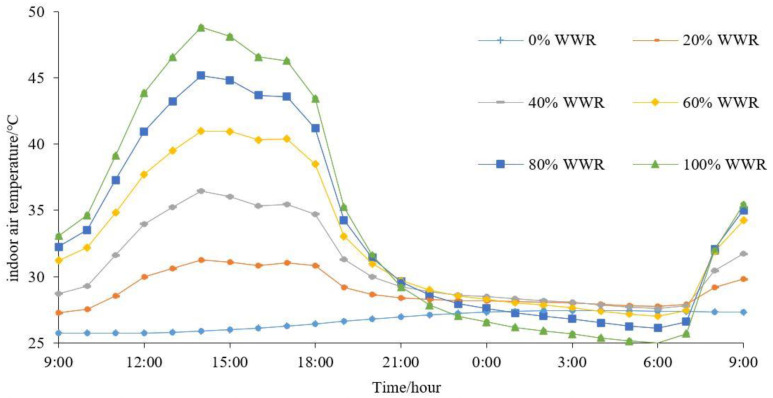
The daily variations of the indoor air temperatures of the six WWRs.

A total of 144 (24 × 6) indoor data were collected, with 24 accounting for the 24 h in the day and 6 accounting for the total number of WWR scenarios. The daily variation of indoor air temperatures in the six WWR cases is presented in Figure 10. Figure 10 indicates that with an increase in WWR, the indoor air temperature increases during daytime and decreases at night.

**Figure 11:**
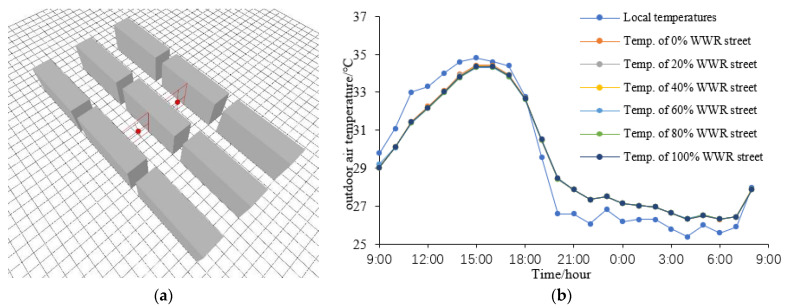
(**a**) Temperature collected points; (**b**) The mean outdoor temperatures.

Additionally, the air temperature at the two points (the three-dimensional centers of the street canyons) are collected and presented in Figure 11a. Their average values are employed to represent the mean air temperature around the buildings investigated in this study.

The mean air temperatures around the investigated building of the six scenarios are shown in Figure 11b. Figure 11b confirms that the air temperatures around the building is significantly different from that of the local air temperatures recorded by the official weather station, which is an effect of the urban microclimate. Figure 11b also shows that the calculation of energy demand by TRNSYS has significant defects, because, in the traditional calculation, the outdoor temperature is not accurate enough. 

### 3.2. Impacts of WWRs on Indoor Cooling Energy Demand

**Figure 12:**
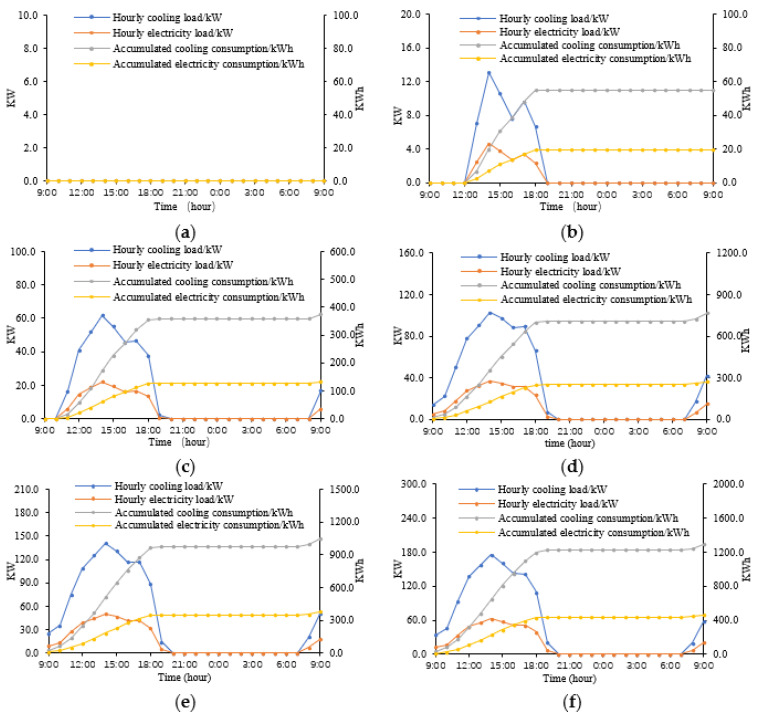
The cooling energy demands of the six scenarios: (**a**) Energy demand of 0% WWR building; (**b**) Energy demand of 20% WWR building; (**c**) Energy demand of 40% WWR building; (**d**) Energy demand of 60% WWR building; (**e**) Energy demand of 80% WWR building; (**f**) Energy demand of 100% WWR building.

The cooling demands and the corresponding electricity demands in the six WWR cases are presented in Figure 12, as calculated by TRNSYS. The subgraphs a, b, c, d, e, and f respectively indicate the scenarios of 0% WWR, 20% WWR, 40% WWR, 60% WWR, 80% WWR, and 100% WWR. In each subgraph, the blue line represents the hourly cooling load, the red line is the hourly electricity load, the grey line, the accumulated cooling consumption, and the yellow line indicates the accumulated electricity consumption.

Figure 12 indicates that on a typical summer day in southern Hunan, the cooling energy demands vary greatly in the six WWR scenarios. Specifically, in a typical six-story residential building with 24 families, in order to maintain an acceptable indoor temperature (below 30 °C), the total cooling demands throughout the day are as follows: 0 KW·h (0% WWR), 54.8 KW·h (20% WWR), 374.3 KW·h (40% WWR), 764.7 KW·h (60% WWR), 1048.6 KW·h (80% WWR), and 1293.2 KW·h (100% WWR). Similarly, the TRNBuild model also calculated the electricity demands to keep the indoor air temperature below 30 °C. Correspondingly, the electricity demands are as follows: 0 KW·h (0% WWR), 19.6 KW·h (20% WWR), 133.7 KW·h (40% WWR), 273.1 KW·h (60% WWR), 374.5 KW·h (80% WWR), and 461.9 KW·h (100% WWR). The analytical results indicate that, on a summer day, the average electricity demand for indoor cooling in a typical residential building increases by about 100 KW·h for each 20% increase in WWR.

## 4. Conclusions

Window-to-wall ratio is an important spatial parameter in urban planning and building science. Urban planning scholars have investigated its impacts on the outdoor thermal environment using CFD-based models, but these investigations did not go deep into its impact on indoor energy consumption. On the other hand, building science scholars have focused on the influence of WWR on energy consumption using transient systems simulation programs. Nevertheless, they only investigated WWR in a single building without considering the impact of the microclimate created by the urban environment.

This study has attempted to couple the research methods used by urban planning scholars and building science scholars. The indoor and outdoor meteorological parameters simulated by Envi-met have been used as the input data for TRNSYS to improve the accuracy of its predictions. This is also useful for the assessment of energy consumption at the urban planning stage.

The analytical results reveal that, on a typical summer day in southern Hunan, an increase in WWR is not beneficial in terms of energy savings. In a six-story residential building with 24 households, in order to avoid indoor air temperatures above 30 °C, the cooling electricity required for the buildings with 0% WWR, 20% WWR, 40% WWR, 60% WWR, 80% WWR, and 100% WWR is 0 KW·h, 19.6 KW·h, 133.7 KW·h, 273.1 KW·h, 374.5 KW·h, and 461.9 KW·h, respectively.

Existing studies have already explored the influences of WWR on cooling or heating energy demands. Those studies have ignored the impacts of urban microclimates on building cooling demand and investigated WWR in single buildings, thus causing inaccuracies in the energy demand predictions.

Although this study has proposed a modified method, which gives more accurate evaluation of WWR impact on cooling energy demand, there are still some limitations to be further explored.

(1) In this study, the windows are considered closed. How would open windows affect the results of this study?
(2) The buildings modeled here are north–south oriented, and the windows have no shading devices or glass coatings. What would be the effects of window orientation, shading devices, and glass coatings on the results of this study?

These are questions that could be further investigated in future studies.

## Figures and Tables

**Table 1:** The properties of the glass and concrete.

Elements	Parameters	Value
Concrete	Thickness (mm)	310.00
	Absorption (Frac)	0.50
	Transmission (Frac)	0.00
	Reflection (Frac)	0.50
	Emissivity (Frac)	0.90
	Specific heat (J/(kg·K)	850.00
	Thermal conductivity (W/(m·K)	1.60
	Density (kg/m^3^)	2220.00
Clear float glass	Thickness (mm)	20.00
	Absorption (Frac)	0.05
	Transmission (Frac)	0.90
	Solar heat gain coefficient	0.80
	Thermal transmittance (U-value) (W/m^2^·K)	5.62
	Reflection (Frac)	0.05
	Emissivity (Frac)	0.90
	Specific heat (J/(kg·K)	750.00
	Thermal conductivity (W/(m·K)	1.05
	Density (kg/m^3^)	2500.00

## Data Availability

Data are available upon request.

## References

[1] Zhang Y., He C.-Q., Tang B.-J., Wei Y.-M. (2015). China’s energy consumption in the building sector: A life cycle approach. Energy Build..

[2] Mi Z., Zhang Y., Guan D., Shan Y., Liu Z., Cong R., Yuan X.-C., Wei Y.-M. (2016). Consumption-based emission accounting for Chinese cities. Appl. Energy.

[3] Yang L., Yan H., Lam J.C. (2014). Thermal comfort and building energy consumption implications—A review. Appl. Energy.

[4] Chen S., Yoshino H., Li N. (2010). Statistical analyses on summer energy consumption characteristics of residential buildings in some cities of China. Energy Build..

[5] Li D.H., Yang L., Lam J.C. (2012). Impact of climate change on energy use in the built environment in different climate zones—A review. Energy.

[6] Li J., Yang L., Long H. (2018). Climatic impacts on energy consumption: Intensive and extensive margins. Energy Econ..

[7] Wang X., Chen D., Ren Z. (2010). Assessment of climate change impact on residential building heating and cooling energy requirement in Australia. Build. Environ..

[8] Chokhachian A., Perini K., Giulini S., Auer T. (2020). Urban performance and density: Generative study on interdependencies of urban form and environmental measures. Sustain. Cities Soc..

[9] Alghoul S.K., Rijabo H.G., Mashena M.E. (2017). Energy consumption in buildings: A correlation for the influence of window to wall ratio and window orientation in Tripoli, Libya. J. Build. Eng..

[10] Tong S., Wong N.H., Tan E., Jusuf S.K. (2019). Experimental study on the impact of facade design on indoor thermal environment in tropical residential buildings. Build. Environ..

[11] Shao N., Zhang J., Ma L. (2017). Analysis on indoor thermal environment and optimization on design parameters of rural residence. J. Build. Eng..

[12] Lu Y., Yang Z., Yu J., Chen B., Zhong K. (2021). Development of a second-order dynamic model for quantifying impact of thermal mass on indoor thermal environment. J. Build. Eng..

[13] Sayadi S., Hayati A., Salmanzadeh M. (2021). Optimization of Window-to-Wall Ratio for Buildings Located in Different Climates: An IDA-Indoor Climate and Energy Simulation Study. Energies.

[14] Marino C., Nucara A., Pietrafesa M. (2017). Does window-to-wall ratio have a significant effect on the energy consumption of buildings? A parametric analysis in Italian climate conditions. J. Build. Eng..

[15] Ma P., Wang L.-S., Guo N. (2015). Maximum window-to-wall ratio of a thermally autonomous building as a function of envelope U-value and ambient temperature amplitude. Appl. Energy.

[16] Li J., Zheng B., Chen X., Zhou Y., Rao J., Bedra K.B. (2020). Research on Annual Thermal Environment of Non-Hvac Building Regulated by Window-to-Wall Ratio in a Chinese City (Chenzhou). Sustainability.

[17] Alwetaishi M., Taki A. (2020). Investigation into energy performance of a school building in a hot climate: Optimum of window-to-wall ratio. Indoor Built Environ..

[18] Troup L., Phillips R., Eckelman M.J., Fannon D. (2019). Effect of window-to-wall ratio on measured energy consumption in US office buildings. Energy Build..

[19] Aburas M., Soebarto V., Williamson T., Liang R., Ebendorff-Heidepriem H., Wu Y. (2019). Thermochromic smart window technologies for building application: A review. Appl. Energy.

[20] Liu Z., Wu D., Li J., Yu H., He B. (2019). Optimizing building envelope dimensions for passive solar houses in the Qinghai-Tibetan region: Window to wall ratio and depth of sunspace. J. Therm. Sci..

[21] Alwetaishi M., Benjeddou O. (2021). Impact of Window to Wall Ratio on Energy Loads in Hot Regions: A Study of Building Energy Performance. Energies.

[22] Toparlar Y., Blocken B., Maiheu B., Van Heijst G. (2017). A review on the CFD analysis of urban microclimate. Renew. Sustain. Energy Rev..

[23] Tang L., Nikolopoulou M., Zhao F.-Y., Zhang N. (2012). CFD modeling of the built environment in Chinese historic settlements. Energy Build..

[24] Khaled F., Aly A.M., Elshaer A. (2021). Computational Efficiency of CFD Modeling for Building Engineering: An Empty Domain Study. J. Build. Eng..

[25] Mittal H., Sharma A., Gairola A. (2019). Numerical simulation of pedestrian level wind flow around buildings: Effect of corner modification and orientation. J. Build. Eng..

[26] Hassan A.M., ELMokadem A.A., Megahed N.A., Eleinen O.M.A. (2020). Urban morphology as a passive strategy in promoting outdoor air quality. J. Build. Eng..

[27] Tien P.W., Calautit J.K. (2019). Numerical analysis of the wind and thermal comfort in courtyards “skycourts” in high rise buildings. J. Build. Eng..

[28] Jaber S., Ajib S. (2011). Optimum, technical and energy efficiency design of residential building in Mediterranean region. Energy Build..

[29] Buratti C., Belloni E., Palladino D. (2014). Evolutive Housing System: Refurbishment with new technologies and unsteady simulations of energy performance. Energy Build..

[30] Catalina T., Iordache V., Caracaleanu B. (2013). Multiple regression model for fast prediction of the heating energy demand. Energy Build..

[31] Perini K., Chokhachian A., Dong S., Auer T. (2017). Modeling and simulating urban outdoor comfort: Coupling ENVI-Met and TRNSYS by grasshopper. Energy Build..

[32] El Bat A.M.S., Romani Z., Bozonnet E., Draoui A. (2021). Thermal impact of street canyon microclimate on building energy needs using TRNSYS: A case study of the city of Tangier in Morocco. Case Stud. Therm. Eng..

[33] Vallati A., Vollaro A.D.L., Golasi I., Barchiesi E., Caranese C. (2015). On the impact of urban micro climate on the energy consumption of buildings. Energy Procedia.

[34] Assimakopoulos M., Mihalakakou G., Flocas H. (2007). Simulating the thermal behaviour of a building during summer period in the urban environment. Renew. Energy.

[35] Zheng X., Wei C., Qin P., Guo J., Yu Y., Song F., Chen Z. (2014). Characteristics of residential energy consumption in China: Findings from a household survey. Energy Policy.

[36] Li C., Xia W., Chai Y. (2021). Delineation of an Urban Community Life Circle Based on a Machine-Learning Estimation of Spatiotemporal Behavioral Demand. Chin. Geogr. Sci..

[37] Feng W., Zou L., Gao G., Wu G., Shen J., Li W. (2016). Gasochromic smart window: Optical and thermal properties, energy simulation and feasibility analysis. Sol. Energy Mater. Sol. Cells.

[38] Tsoka S., Tsikaloudaki A., Theodosiou T. (2018). Analyzing the ENVI-met microclimate model’s performance and assessing cool materials and urban vegetation applications—A review. Sustain. Cities Soc..

[39] Zhai Z., Chen Q.Y. (2003). Solution characters of iterative coupling between energy simulation and CFD programs. Energy Build..

[40] Yoshino H., Yoshino Y., Zhang Q., Mochida A., Li N., Li Z., Miyasaka H. (2006). Indoor thermal environment and energy saving for urban residential buildings in China. Energy Build..

[41] Li J., Zheng B., Shen W., Xiang Y., Chen X., Qi Z. (2019). Cooling and energy-saving performance of different green wall design: A simulation study of a block. Energies.

[42] Liao J., Tan X., Li J. (2021). Evaluating the vertical cooling performances of urban vegetation scenarios in a residential environment. J. Build. Eng..

[43] Li J., Zheng B., Chen X., Qi Z., Bedra K.B., Zheng J., Li Z., Liu L. (2021). Study on a full-year improvement of indoor thermal comfort by different vertical greening patterns. J. Build. Eng..

[44] Carnielo E., Zinzi M. (2013). Optical and thermal characterisation of cool asphalts to mitigate urban temperatures and building cooling demand. Build. Environ..

[45] Bruse M. (2004). ENVI-met 3.0: Updated Model Overview.

[46] Chen Y.-C., Lin T.-P., Matzarakis A. (2014). Comparison of mean radiant temperature from field experiment and modelling: A case study in Freiburg, Germany. Theor. Appl. Climatol..

[47] Salata F., Golasi I., de Lieto Vollaro R., de Lieto Vollaro A. (2016). Urban microclimate and outdoor thermal comfort. A proper procedure to fit ENVI-met simulation outputs to experimental data. Sustain. Cities Soc..

[48] Daemei A.B., Azmoodeh M., Zamani Z., Khotbehsara E.M. (2018). Experimental and simulation studies on the thermal behavior of vertical greenery system for temperature mitigation in urban spaces. J. Build. Eng..

[49] Yang W., Wong N.H., Zhang G. (2013). A comparative analysis of human thermal conditions in outdoor urban spaces in the summer season in Singapore and Changsha, China. Int. J. Biometeorol..

[50] Yang W., Wong N.H. (2013). Field study of human thermal perception in urban parks in Singapore. Int. J. Sustain. Build. Technol. Urban Dev..

[51] Ng E., Cheng V. (2012). Urban human thermal comfort in hot and humid Hong Kong. Energy Build..

[52] Liu W., Zhang Y., Deng Q. (2016). The effects of urban microclimate on outdoor thermal sensation and neutral temperature in hot-summer and cold-winter climate. Energy Build..

[53] Ibanez M., Lázaro A., Zalba B., Cabeza L.F. (2005). An approach to the simulation of PCMs in building applications using TRNSYS. Appl. Therm. Eng..

